# [^18^F]FDG uptake of the normal spinal cord in PET/MR imaging: comparison with PET/CT imaging

**DOI:** 10.1186/s13550-020-00680-8

**Published:** 2020-08-06

**Authors:** Marco Aiello, Vincenzo Alfano, Elena Salvatore, Carlo Cavaliere, Marco Picardi, Roberta Della Pepa, Emanuele Nicolai, Andrea Soricelli, Alessandra Vella, Marco Salvatore, Mario Mascalchi

**Affiliations:** 1 IRCCS SDN, Naples, Italy; 2 Department of Neuroscience, University of Naples Federico II, Naples, Italy; 3 Department of Clinical Medicine and Surgery, Hematology Section, I, Naples, Italy; 4 Nuclear Medicine, «Le Scotte» Hospital of Siena, Siena, Italy; 5 «Mario Serio» Department of Experimental and Clinical Biomedical Sciences, University of Florence, Florence, Italy

**Keywords:** FDG, PET/MRI, Spinal cord, SUV

## Abstract

**Background:**

The lack of visualization of the spinal cord hinders the evaluation of [^18^F]Fluoro-deoxy-glucose (FDG) uptake of the spinal cord in PET/CT. By exploiting the capability of MRI to precisely outline the spinal cord, we performed a retrospective study aimed to define normal pattern of spinal cord [^18^F]FDG uptake in PET/MRI.

**Methods:**

Forty-one patients with lymphoma without clinical or MRI signs of spinal cord or bone marrow involvement underwent simultaneous PET and MRI acquisition using Siemens Biograph mMR after injection of 3.5 MBq/kg body weight of [^18^F]FDG for staging purposes. Using a custom-made software, we placed ROIs of 3 and 9 mm in diameter in the spinal cord, lumbar CSF, and vertebral marrow that were identified on MRI at 5 levels (C2, C5, T6, T12, and L3). The SUVmax, SUVmean, and the SUVmax and SUVmean normalized (NSUVmax and NSUVmean) to the liver were measured. For comparison, the same ROIs were placed in PET-CT images obtained immediately before the PET-MRI acquisition following the same tracer injection.

**Results:**

On PET/MRI using the 3 mm ROI, the following average (all level excluding L3) spinal cord median (1st and 3rd quartile) values were measured: SUVmean, 1.68 (1.39 and 1.83); SUVmax, 1.92 (1.60 and 2.14); NSUVmean, 1.18 (0.93 and 1.36); and NSUVmax, 1.27 (1.01 and 1.33). Using the 9 mm ROI, the corresponding values were SUVmean, 1.41 (1.25–1.55); SUVmax, 2.41 (2.08 and 2.61); NSUVmean, 0.93 (0.79 and 1.04); and NSUVmax, 1.28 (1.02 and 1.39). Using the 3 mm ROI, the highest values of PET-MRI SUVmax, SUVmean, NSUVmax, and NSUVmean were consistently observed at C5 and the lowest at T6. Using a 9 mm ROI, the highest values were consistently observed at C5 and the lowest at T12 or T6. The spinal cord [^18^F]FDG-uptake values correlated with the bone marrow uptake at the same level, especially in case of NSUVmax. Comparison with PET-CT data revealed that the average SUVmax and SUVmean of the spinal cord were similar in PET-MRI and PET-CT. However, the average NSUVmax and NSUVmean of the spinal cord were higher (range 21–47%) in PET-MRI than in PET-CT.

**Conclusions:**

Using a whole-body protocol, we defined the maximum and mean [^18^F]FDG uptake of the normal spinal cord in PET/MRI. While the observed values show the expected longitudinal distribution, they appear to be higher than those measured in PET/CT. Normalization of the SUVmax and SUVmean of the spinal cord to the liver radiotracer uptake could help in multi-institutional comparisons and studies.

## Background

Functional imaging of the spinal cord with positron emission tomography (PET) represents a distinctive challenge due to the its small size, the relatively low resolution of PET images, and contamination from vertebral bone marrow [1]. Studies published so far utilized PET/CT and the metabolic tracer [^18^F]Fluoro-deoxy-glucose (FDG) according to a whole-body [2, 3] or dedicated spinal protocol [4].

The more reliable measurement of [^18^F]FDG uptake in PET/CT studies of normal and diseased spinal cord is uncertain [1]. Two approaches have been utilized. One entails measurement of the standardized uptake value (SUV) of the normal or affected spinal cord per se that is usually sampled with a region of interest (ROI) encompassing the entire spinal canal due to lack of the spinal cord definition and taking care to exclude the vertebral bone. The other implies measurement of the ratio of SUV to background using the activity of the normal spinal cord, lumbar thecal sac, vertebral marrow, or liver as a reference [2–4]. Moreover, due to inclusion of the surrounding CSF in the spinal cord ROI sampling in PET/CT, the spinal cord SUVmax, that is assumed to reflect the higher metabolic activity of the cord central gray matter, was usually preferred to SUVmean in PET/CT studies of spinal cord diseases.

To tackle the problem of lack of visualization of the spinal cord, which hinders evaluation of [^18^F]FDG uptake of the cord in PET/CT, it is now possible to take advantage of the introduction of hybrid PET/MR scanners. In fact, they allow to simultaneously acquire both metabolic (through PET imaging) and morphological data with excellent tissue contrast (via MRI) [5, 6]. Moreover, simultaneous hybrid systems can also overcome the problem of the PET and MR coregistration, since PET and MR images (of the same anatomical district) are obtained at the same time and can therefore ideally coregistered [7]. Moreover, the visualization of the spinal cord contour in PET/MRI enables measurement of the SUV across the entire ROI (SUVmean) that might represent an additional useful tool in certain disease conditions.

The aim of this study was to demonstrate the feasibility of PET/MR imaging of the spinal cord. In particular, by exploiting the capability of MRI to precisely outline the spinal cord, we performed a semi-automated ROI analysis of the [^18^F]FDG uptake in PET/MRI in adults without evidence of the spinal cord or bone marrow abnormalities. The [^18^F]FDG uptake values in PET/MRI were compared to the data on PET/CT imaging of the spinal cord in the same patient population.

## Material and methods

This is a retrospective study performed in a single center where two hybrid PET scanners, one PET/CT, and one PET/MRI operate. Scans were acquired after 6 h of fasting. Body weight, height, and glucose levels were measured. After intravenous injection of an activity of 3.5 MBq of FDG/kg body weight, patients were resting for 60 min. During whole body acquisition, the patient was placed supine in the scanner so that he or she was comfortable, and motion could be minimized during the acquisition. Patients were sequentially examined with PET/CT (10 min duration) and PET/MRI (60 min duration), exploiting a single injection of radiotracer, thus avoiding delivery of additional ionizing radiations as compared to PET/CT alone; mean interval between the start of PET/CT and PET/MRI was 55 min (± 27 min); median was 45 min. The patient provided a written informed consent before the injection and acquisition.

### Patients selection

Forty-seven patients with early stage lymphoma prior to treatment and not in a hyperacute phase were retrospectively enrolled. The following exclusion criteria were applied: (1) presence of motion between PET and CT datasets with failed/poor coregistration and (2) known spinal canal or cord diseases based on existing imaging and chart review. Finally, 41 patients were selected (20 men and 21 women) with a mean age of 46.9 ± 18.7 years.

### [^18^F]FDG PET/CT acquisition

A Discovery IQ hybrid (GE Healthcare, Milwaukee, WI, USA) PET-CT scanner (spatial resolution at 1 cm, 4.5/4.5 mm as per NEMA definition) was used with tube voltage, 140 kVp; pitch, 0.94; and voxel size of reconstructed PET image, 4 × 4 × 4 mm^3^. PET data were acquired using the three-dimensional (3D) mode with a fixed scan duration of 2 min per bed position. Emission data were corrected for random coincidences, dead time, and scatter. CT data were used for a measured attenuation correction and assistance in anatomic localization of FDG. PET emission data were reconstructed with ordered subset-expectation maximization (OSEM) algorithm (21 subsets, 4 iterations) and post-filtered with a three-dimensional isotropic Gaussian of 4 mm full width at half maximum.

### [^18^F]FDG PET/MRI acquisition

A 3T mMR Siemens (Erlangen, Germany) Biograph hybrid PET-MR system (spatial resolution at 1 cm, 4.3/4.3 mm as per NEMA definition) was used [8], with PET images (voxel size 4.17 × 4.17 × 2.03 mm^3^) acquired simultaneously (5 min per bed position) with whole body MRI T2 HASTE sequence (TR, 1400; TE, 87; slice thickness, 6 mm; field of view, 380 × 380) and automatically corrected for tissue attenuation with the attenuation maps generated by using tissue segmentations from Dixon MRI sequence [6] (first TE, 1.23 ms; second TE, 2.46 ms; TR, 3.96 ms; voxel size, 4.1 × 2.6 × 3.1 mm; field of view, 500 × 312). Emission data were corrected for random coincidences, dead time, scatter, and attenuation. PET data were reconstructed with a 3D attenuation weighted ordered-subsets expectation maximization iterative reconstruction algorithm (AW OSEM 3D) with three iterations and 21 subsets, Gaussian smoothing 4 mm full width at half maximum.

### ROI and statistical analyses

**Fig. 1:**
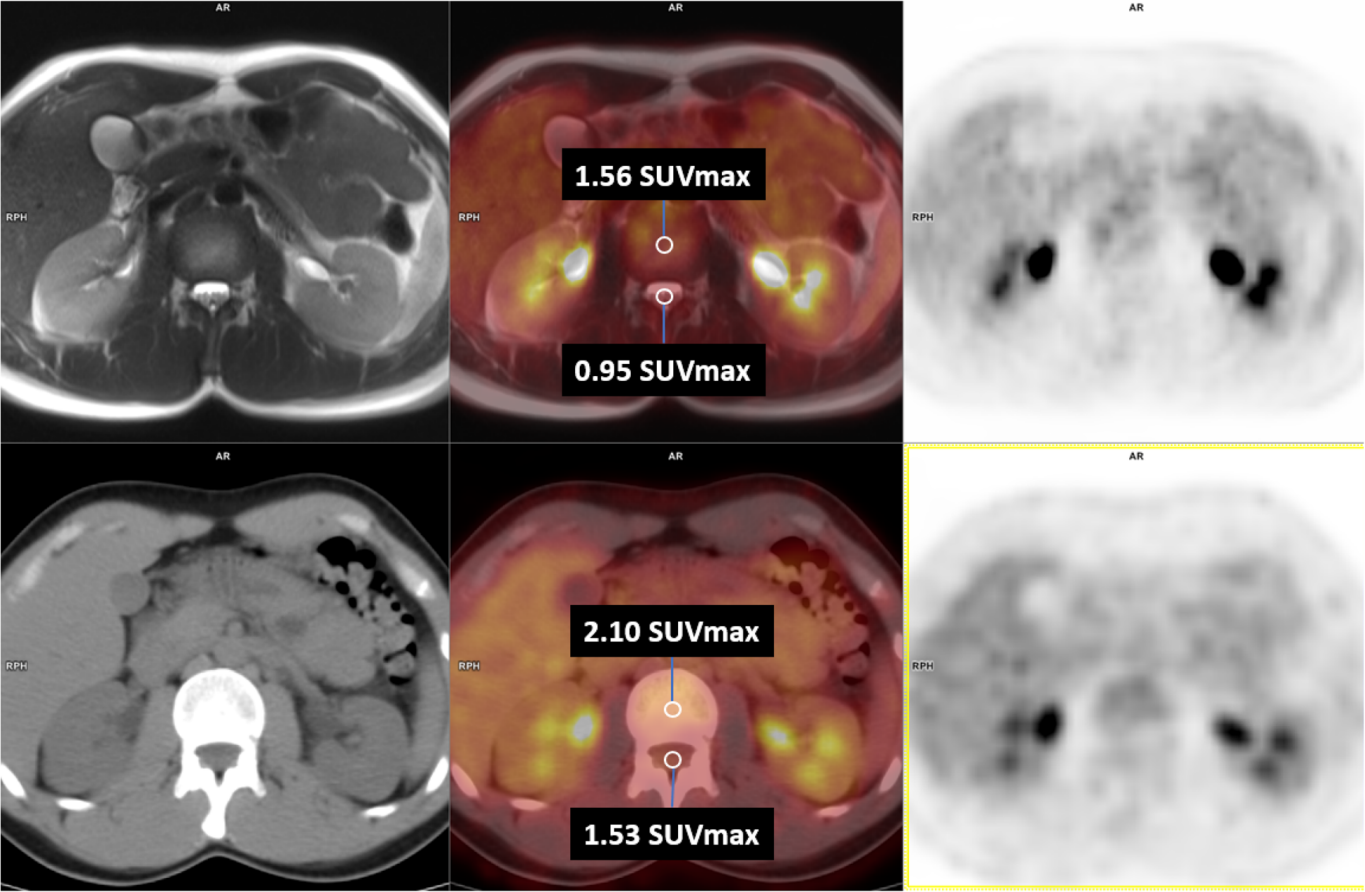
Example of a 3 mm radius ROI at T12 level. The ROI was centered on the spinal cord and the corresponding vertebral body bone marrow. Top row from left to right (MRI, PET/MRI, PET); bottom row from left to right (CT, PET/CT, PET)

We used a custom-made software, named SMART anatomical LABelling (SMARTLAB) tool for medical imaging datasets, based on the Syngo.Frontier platform (Siemens, Germany), to place fiducial points in PET/MRI and PET/CT images. Fiducial points in the spinal cord, lumbar CSF, and vertebral marrow were identified on MRI and CT at five levels (C2, C5, T6, T12, and L3). An additional fiducial point was placed in the liver, on the 6th hepatic segment [9], avoiding blood vessels. The space containing the fiducial point was imported on a MATLAB preprocessing pipeline which includes the re-slicing and coregistering of PET/MRI and PET/CT images on the space of the fiducial points. From the fiducial point, a circular ROI of 3 mm radius and a spherical volume of interest (VOI) of 9 mm radius were drawn for each level. The choice of the 3 mm ROI and 9 mm VOI was made in order to detect the SUV on a single slice for the ROI, and on at least 2 slices for the VOI, considering the 6 mm slice thickness of whole body T2 haste MR sequence. The ROI/VOI was centered on the spinal cord and the corresponding vertebral body bone marrow (Fig. 1). Then, the SUVmax and the SUVmean were measured. In addition, as suggested by Marini et al. [4], a reference ROI in the liver was measured in both PET/MRI and PET/CT and enabled to calculate the normalized SUVmax (NSUVmax) and the normalized SUVmean (NSUVmean). To test the coherence of the quantitative results of this study, we divided the subjects in two subgroups, namely, those without and those with increased uptake at visual examination in the cervical enlargement (Group 1 and Group 2, respectively) and then compare the spinal cord SUVmax values in PET/MRI and PET/CT between the two subgroups.

A preliminary Shapiro-Wilk test showed that the data distribution was not normal. Accordingly, we adopted non-parametric tests for statistical analyses. We used Wilcoxon test to compare the spinal cord and bone marrow [^18^F]FDG uptake in PET/MRI and PET/CT and the Spearman rank correlation test to evaluate the correlation between spinal cord and bone marrow uptake at the same spinal level. Bonferroni correction for multiple comparison was applied (*p* value < 0.01).

## Results

Only the NSUVmax of the spinal cord in 3 or 9 mm ROI/VOIs was significantly different between PET/MRI and PET/CT and consistently higher in the former (Table 1). The SUVmax, SUVmean, and NSUVmean in the spinal cord are reported in supplementary materials (Table S1).

**Table 1:** Median, 1st and 3rd quartile of NSUVmax values measured in the spinal cord in both PET-CT and PET-MR, with 3 mm ROI and 9 mm VOI

Spinal cord	NSUVmax in 3 mm ROI										
	PET-CT					PET-MR					*p*
	q1	Median	q3	Mean	SD	q1	Median	q3	Mean	SD	Wilcoxon
**C2**	0.69	0.79	0.93	0.82	0.23	0.98	1.14	1.37	1.27	0.69	< 0.0001
**C5**	0.68	0.85	0.94	0.83	0.21	1.15	1.39	1.60	1.48	1.07	< 0.0001
**T6**	0.55	0.68	0.79	0.7	0.23	0.84	0.95	1.06	1.12	0.80	< 0.0001
**T12**	0.63	0.83	1.04	0.85	0.28	0.88	1.12	1.35	1.22	0.79	< 0.0001
**CSF L3**	0.42	0.50	0.62	0.54	0.20	0.43	0.56	0.73	0.68	0.64	n.s.
	**NSUVmax in 9 mm VOI**										
	**PET-CT**					**PET-MR**					*p*
	q1	Median	q3	Mean	SD	q1	Median	q3	Mean	SD	Wilcoxon
**C2**	0.67	0.82	0.98	0.88	0.33	0.88	1.14	1.31	1.20	0.61	< 0.001
**C5**	0.74	0.84	0.9	0.85	0.23	1.02	1.34	1.53	1.39	0.80	< 0.0001
**T6**	0.64	0.81	0.95	0.87	0.38	0.96	1.17	1.38	1.36	0.82	< 0.0001
**T12**	0.73	0.90	1.03	0.92	0.29	0.86	1.07	1.29	1.16	0.60	< 0.001
**CSF L3**	0.57	0.69	0.84	0.76	0.34	0.62	0.73	0.84	0.79	0.49	n.s.

A significant difference between PET/MRI and PET/CT was also observed for the SUVmax and NSUVmax in the bone marrow using both 3 mm and 9 mm ROI/VOI with consistently higher values in the former (Table 2). The SUVmean and NSUVmean in the bone marrow are reported in supplementary materials (Table S2), and the average SUVmax and SUVmean of the liver, used as reference in NSUV values, in PET/MRI and PET/CT is reported in supplementary materials (Table S3).

**Table 2:** Median, 1st and 3rd quartile of SUVmax and NSUVmax values measured in the bone marrow in both PET-CT and PET-MR, with 3 mm ROI and 9 mm VOI

Bone marrow	SUVmax in 3 mm ROI										
	PET-CT					PET-MR					*p*
	q1	Median	q3	Mean	SD	q1	Median	q3	Mean	SD	Wilcoxon
**C2**	1.37	1.64	2.25	1.82	0.70	1.76	2.28	2.90	2.39	0.97	< 0.0001
**C5**	1.38	1.71	2.13	1.83	0.64	1.85	2.37	2.90	2.48	0.88	< 0.0001
**T6**	1.93	2.52	3.18	2.61	0.91	2.32	2.85	3.71	3.18	1.18	< 0.0001
**T12**	1.95	2.53	3.20	2.65	0.90	2.12	2.47	3.63	2.81	1.26	n.s.
**L3**	1.93	2.30	3.01	2.58	0.95	2.11	2.75	3.43	2.98	1.35	< 0.001
	**SUVmax in 9 mm VOI**										
	**PET-CT**					**PET-MR**					*p*
	q1	Median	q3	Mean	SD	q1	Median	q3	Mean	SD	Wilcoxon
**C2**	1.55	2.18	2.53	2.19	0.89	2.07	2.41	3.25	2.71	1.03	< 0.0001
**C5**	1.77	2.15	2.67	2.27	0.69	2.32	2.73	3.71	3.04	1.10	< 0.0001
**T6**	2.26	2.82	3.60	2.94	1.00	2.77	3.31	4.83	3.83	1.47	< 0.0001
**T12**	2.38	2.87	3.72	3.13	1.02	2.50	2.77	3.93	3.24	1.35	n.s.
**L3**	2.30	2.78	3.61	2.99	0.99	2.78	3.46	4.20	3.73	1.43	< 0.0001
**Bone marrow**	**NSUVmax in 3 mm ROI**										
	**PET-CT**					**PET-MR**					*p*
	q1	Median	q3	Mean	SD	q1	Median	q3	Mean	SD	Wilcoxon
**C2**	0.53	0.72	0.99	0.8	0.36	0.96	1.32	1.71	1.69	1.75	< 0.0001
**C5**	0.61	0.74	0.95	0.8	0.28	1.09	1.40	1.75	1.61	1.04	< 0.0001
**T6**	0.86	1.07	1.23	1.15	0.46	1.33	1.73	2.19	2.21	2.18	< 0.0001
**T12**	0.83	1.15	1.33	1.18	0.46	1.22	1.47	1.94	1.97	2.16	< 0.0001
**L3**	0.82	1.08	1.31	1.14	0.43	1.03	1.59	2.06	2.11	2.17	< 0.0001
	**NSUVmax in 9 mm VOI**										
	**PET-CT**					**PET-MR**					*p*
	q1	Median	q3	Mean	SD	q1	Median	q3	Mean	SD	Wilcoxon
**C2**	0.57	0.81	0.93	0.83	0.39	0.93	1.22	1.49	1.47	1.12	< 0.0001
**C5**	0.66	0.80	1.05	0.86	0.30	1.08	1.37	1.79	1.71	1.57	< 0.0001
**T6**	0.81	1.00	1.29	1.11	0.47	1.35	1.56	2.18	2.06	1.63	< 0.0001
**T12**	0.9	1.11	1.36	1.18	0.44	1.14	1.45	1.79	1.73	1.29	< 0.0001
**L3**	0.83	1.07	1.35	1.13	0.39	1.3	1.59	2.19	2.05	1.71	< 0.0001

**Fig. 2:**
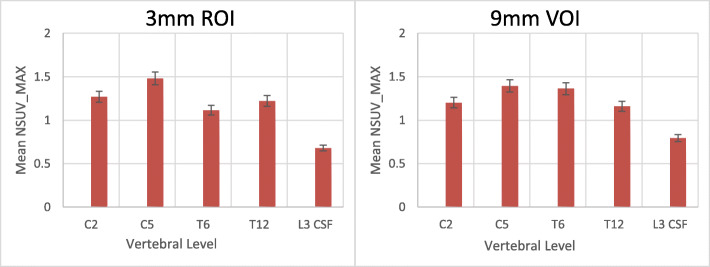
Mean and SD values of the NSUVmax in PET/MRI of the spinal cord at C2, C5, T6, and T12 levels and of CSF at L3

Using the 3 mm ROI in PET/MRI, the highest values of SUVmax, SUVmean, NSUVmax, and NSUVmean of the spinal cord were observed at C5 level and the lowest at T6. Using the 9 mm VOI, the highest values were observed at C5 level, while the lowest values were observed at T12 for SUVmax and NSUVmax and at T6 for SUVmean and NSUVmean. Figure 2 shows the distribution of the spinal cord NSUVmax along the vertebral levels in PET/MRI.

In PET-MRI, the spinal cord [^18^F]FDG-uptake values correlated with bone marrow uptake at the same level, especially in case of NSUVmax (Table 3).

**Table 3:** Spearman correlation between spinal cord and marrow [^18^F]FDG uptake at the same vertebral level for PET-MRI images with 3–9 mm ROI/VOI

ROI = 3 mm	SUVmax		SUVmean		NSUVmax		NSUVmean	
PET-MR	rho	*p*	rho	*p*	rho	*p*	rho	*p*
C2-C2_s	0.00	n.s.	0.07	n.s.	0.28	n.s.	0.37	n.s.
C5-C5_s	0.14	n.s.	0.27	n.s.	0.49	< 0.01	0.60	< 0.0001
T6-T6_s	0.54	< 0.001	0.48	< 0.01	0.74	< 0.0001	0.71	< 0.0001
T12-T12_s	0.40	< 0.01	0.39	n.s.	0.71	< 0.0001	0.73	< 0.0001
L3-L3_s	0.35	n.s.	0.43	< 0.01	0.53	< 0.001	0.58	< 0.0001
**ROI = 9 mm**	**SUVmax**		**SUVmean**		**NSUVmax**		**NSUVmean**	
	rho	*p*	rho	*p*	rho	*p*	rho	*p*
C2-C2_s	0.19	n.s.	0.27	n.s.	0.40	< 0.01	0.52	< 0.001
C5-C5_s	0.44	< 0.01	0.32	< 0.001	0.71	< 0.0001	0.74	< 0.0001
T6-T6_s	0.83	< 0.0001	0.58	< 0.0001	0.87	< 0.0001	0.79	< 0.0001
T12-T12_s	0.60	< 0.0001	0.60	< 0.0001	0.76	< 0.0001	0.80	< 0.0001
L3-L3_s	0.75	< 0.0001	0.48	< 0.01	0.76	< 0.0001	0.64	< 0.0001

The average (computed as values at C2, C5, T6, and T12 level/4) spinal cord SUVmax, SUVmean, and NSUVmean in PET/MRI significantly differed using 3 mm ROI and 9 mm VOI in PET/MRI with higher values in the 3 mm ROI for SUVmean and NSUVmean, while higher values in the 9 mm VOI for SUVmax. Table 4 shows the comparison of the average SUVmax and SUVmean and NSUVmax and NSUVmean of the spinal cord, CSF at L3, and average vertebral bone marrow in PET/MRI and PET/CT in our patients. In general, they were similar, but the average NSUVmax and NSUVmean of the spinal cord were higher (range 21–47%) in PET/MRI than in PET/CT. On the other hand, the SUVmax and SUVmean of CSF at L3 were lower in PET/MRI than in PET/CT.

**Table 4:** Median and 1st and 3rd quartile values of the average spinal cord, CSF at L3 level, and average vertebral bone marrow calculated for each method and ROI/VOI

	PET-MRI 3 mm									PET-CT 3 mm								
	q1	Spinal cord	q3	q1	CSF	q3	q1	Bone marrow	q3	q1	Spinal cord	q3	q1	CSF	q3	q1	Bone marrow	q3
SUVmean	1.39	1.65	1.83	0.55	0.79	1.03	1.93	2.28	2.96	1.38	1.58	1.87	0.81	0.96	1.25	1.61	2.01	2.34
SUVmax	1.60	1.87	2.14	0.75	0.89	1.17	2.15	2.54	3.51	1.59	1.77	2.06	0.94	1.13	1.48	1.74	2.18	2.56
NSUVmean	0.93	1.12	1.36	0.41	0.55	0.67	1.15	1.61	1.98	0.68	0.84	1.21	0.39	0.57	0.83	0.78	0.98	1.58
NSUVmax	1.01	1.23	1.33	0.43	0.56	0.73	1.12	1.51	2.05	0.68	0.78	0.90	0.42	0.50	0.62	0.77	0.95	1.14
	**PET-MRI 9 mm**									**PET-CT 9 mm**								
	q1	**Spinal cord**	q3	q1	**CSF**	q3	q1	**Bone marrow**	q3	q1	**Spinal cord**	q3	q1	**CSF**	q3	q1	**Bone marrow**	q3
SUVmean	1.25	1.37	1.55	0.70	0.82	1.01	1.77	1.99	2.57	1.40	1.47	1.72	0.95	1.09	1.23	1.55	1.89	2.16
SUVmax	2.08	2.31	2.61	1.20	1.37	1.65	2.47	3.02	4.05	1.94	2.27	2.59	1.60	1.82	2.15	2.07	2.55	3.24
NSUVmean	0.79	0.96	1.04	0.46	0.54	0.64	1.04	1.23	1.62	0.63	0.76	1.24	0.47	0.58	1.06	0.73	0.95	2.09
NSUVmax	1.02	1.22	1.39	0.62	0.73	0.84	1.25	1.39	1.80	0.71	0.87	0.93	0.57	0.69	0.84	0.76	0.96	1.17

The results of the subgroups analysis indicated that the Group 1 (with increased spinal cord uptake) and the Group 2 (without increased spinal cord uptake) shared the same trend of the overall group, showing higher median SUVmax values in PET/MRI than PET/CT in both 3 mm ROI and 9 mm VOI (Table S4).

## Discussion

So far, the [^18^F]FDG uptake in normal and diseased spinal cord was evaluated in PET/CT studies [2–4, 10–16]. It is recognized that clinical PET/MRI could be advantageous with respect to PET/CT for assessment of spinal cord diseases [1]. PET/CT studies have shown inconsistent results about the impact of gender, age, body mass index, and blood glucose on SUVmax of the spinal cord [3, 10, 11, 14, 17]. However, in pediatric population, [^18^F]FDG uptake of the spinal cord increased with body weight [14].

We are aware of several technical and methodological differences when the average SUV values we measured in PET/MRI are compared with those measured in PET/CT by us (and others). We administered an activity of 3.5 MBq/kg body weight that is lower to those used in prior [^18^F]FDG/PET studies (4.8–5.2 MBq/kg of body weight [4]). However, while a lower signal to noise ratio can result from lower activity, no effect on SUV is expected [18]. In our protocol, we subsequently acquired PET/CT and PET/MRI. While this may imply lower PET values in PET/MRI, the duration of acquisition was reported not to affect SUVmax values in case of spinal cord tumors [19] and healthy tissue considering that the time activity curve of normal organs (liver and spinal cord) has a slow and linear decay over time [20, 21].

Our method for ROI computation of the spinal cord [^18^F]FDG uptake values in PET/CT was different from that employed in prior PET/CT studies. In fact, we coregistered in PET/CT the ROIs that we drew on MRI taking advantage of the spinal cord direct visualization. In prior PET/CT studies, the spinal cord uptake refers to the SUVmax of a ROI that is drawn along the contour of the spinal canal with care not to include the cortical vertebral bone. This notwithstanding, Patel et al. [3] emphasized the need of correcting for bone marrow contamination when reporting [^18^F]FDG uptake of the spinal cord. Therefore, our spinal cord ROI annotation procedure could be more accurate with respect to prior studies.

All the above differences considered, it is noteworthy that the SUVmax values of the normal spinal cord we measured in PET/CT as at each spinal level or on average over the entire spinal cord were similar to those measured in other PET/CT studies [3, 4, 10, 11, 14, 17]. This notwithstanding, we generally measured higher average [^18^F]FDG uptake values (computed as values at levels C2, C5, T6, and T12 /4) of the spinal cord in PET/MRI than in PET/CT. Moreover, it is conceivable that if a direct PET/MRI acquisition could have been performed without prior PET/CT, [^18^F]FDG uptake values of the spinal cord in PET/MRI in our patients could even be higher than those we measured.

Remarkably, the longitudinal distribution of SUVmax and NSUVmax of the spinal cord we measured in PET/MRI is in line with that previously reported in PET/CT [2, 3, 10, 14, 17]. In particular, using 3 mm ROI, we observed the highest SUVmax values at vertebral level C5 and the lowest values at vertebral level T6. This feature has been attributed to the variable amount of nervous tissue and in particular of the metabolically more active central gray matter in the spinal cord [2, 17]. In fact, the cross-sectional area of the spinal cord decreases from C1 to the conus medullaris with the exception of cervical (from C3 to T2 vertebral body) and lumbar (from the T9 to T12) enlargements, where the transverse diameter increases due to the relative expansion of the gray and white matter that is associated with a greater number of neurons correlated to upper and lower limbs sensory and motor functions. In particular, the C4 to T1 spinal cord neural metamers, corresponding to the vertebral body level from C3 to T2, show a mean cross-sectional area of gray matter from 7.8 to 10.7 mm^2^, and the L3 to S1 spinal cord neural metamers corresponding to vertebral body level from T9–T10 to L1–L2 show a mean cross-sectional of gray matter of 13.2 to 16.7 mm. Differently, the remainder thoracic spinal cord neural metamers show a range from 3.7 to 5.6 mm^2^ of mean cross-section of the gray matter [22, 23]. Accordingly, the SUVmax of the [^18^F]FDG uptake of the spinal cord would reflect different segmental (metameric) levels of specialization and demands with higher values in segments involved in sensory and motion functions of the limbs and lower values in thoracic segments mainly involved in the functions of thoracoabdominal visceral organs and sensory and motion functions of the trunk.

The significant correlation between spinal cord and bone marrow [^18^F]FDG uptake values we observed in both PET/MRI and PET/CT is in line with the PET/CT data by Patel et al. [3]. A distinct advantage provided by PET/MRI as compared to PET/CT is the possibility of reliably measuring the SUVmean, namely, the mean [^18^F]FDG uptake of the cross-sectional area of the spinal cord that is not visible in PET/CT. As expected, inclusion of the cord white matter with its lower metabolism/glucose consumption in the ROI implies lower SUVmean values as compared to the SUVmax values which essentially reflects the higher metabolism/glucose consumption of the central gray matter. However, SUVmean and NSUVmean may be of potential interest for spinal cord diseases affecting predominantly the cord white matter as multiple sclerosis, Friedreich’s ataxia, and VitB2 deficiency.

The longitudinal distribution of SUVmean and NSUVmean we observed closely matches that of SUVmax and NSUVmax. So far, the [^18^F]FDG uptake in PET/CT was measured in spinal cord tumors that are rare conditions in which a pattern of variably increased [^18^F]FDG uptake was reported [12, 17]. [^18^F]FDG uptake in PET/CT of the spinal cord has also been applied to the evaluation of inflammatory myelopathies, including multiple sclerosis, neurosarcoidosis, and of amyotrophic lateral sclerosis [12, 13]. While a pattern of decreased or increased [^18^F]FDG uptake was observed in inflammatory myelopathies and neurosarcoidosis, presumably reflecting varying delay between disease onset and time of PET and possible interference with steroid therapy [12, 13], in case of amyotrophic lateral sclerosis elevated [^18^F]FDG uptake values were reported [24]. Additional potential diseases suitable to be evaluated with PET of the spinal cord include trauma, post-radiation myelopathy, vitamin B12 deficiency myelopathy, and some neurodegenerative diseases which primarily affect the spinal cord as Friedreich’s ataxia and hereditary spastic paraplegia. We anticipate that in these conditions both SUVmax and SUVmean could provide valuable information because the cord white matter is frequently more affected than the central gray matter.

An issue addressed in the present study was the possible effect of normalizing the spinal cord SUV max and mean values to the liver [^18^F]FDG uptake. This type of normalization was originally proposed by Marini et al. [4] for the spinal cord SUVmax in order to pool data provided by two PET/CT scanners. In our instance, the normalization was justified by the need to overcome possible differences in [^18^F]FDG uptake between PET/CT and PET/MRI due to the time interval between the two acquisitions and the tracer decay. Remarkably, all the results we obtained by analyzing SUVmax and SUVmean values in PET/MRI were consistent when the data were normalized to the liver, considering the different decay rate between the spinal cord and the liver, with a quicker one for the liver. This suggests that liver normalization might improve stability of results and might be useful for multi-center studies.

We recognize the following limitations of our study.

We evaluated the spinal cord [^18^F]FDG uptake in a population of adult subjects with lymphoma rather than of healthy subjects. However, the patients we enrolled had neither neurologic symptoms and signs nor MRI evidence of any spinal cord or marrow abnormality. Moreover, they were examined for initial staging purposes and had not received yet chemo or radiation therapies. The sequential acquisition of PET/CT and PET/MRI could have affected the spinal cord uptake values in the PET/MRI. Future PET/MRI studies of neurological conditions could avoid preliminary PET/CT acquisition that was requested by Health Authorities in Italy at the time of the present study. In addition, it is worth mentioning here that current MR-based AC techniques, such as the one used in the present work, produce slightly biased estimates relative to CT-based AC [6, 25]. Therefore, the specific impact on SUV quantification of the spine and spinal cord should be evaluated and taken into account in further studies.

We did not control for factors that in some prior studies were reported to potentially modify the [^18^F]FDG uptake such as lean body mass, body surface area, serum glucose, insulin, free fatty acid, and exact time to acquisition. However, these factors are generally considered crucial in [^18^F]FDG repeat studies and not in cross-sectional studies like ours.

Finally, we used a whole-body protocol instead than a dedicated spinal MRI-PET. The latter could allow better morphologic definition of the spinal cord with improved coregistration and especially correlation with sequences for volumetric and microstructural assessment of the spinal cord. Of course, the combination of metabolic information from [^18^F]FDG PET with macro and microstructural information with high spatial resolution from MRI in the same examination will represent a valuable tool for improved understanding of the physiopathology of spinal cord diseases and might constitute a valuable surrogate marker for future trials. Also, exploration of other radiotracers than [^18^F]FDG may expand the role of spinal cord PET.

## Conclusion

In conclusion, using a whole-body protocol, we defined the maximum and mean [^18^F]FDG uptake of the normal spinal cord in PET/MRI. These values were higher than those measured in PET/CT so widening the dynamic range of this metabolic measurement. The SUV values showed the expected longitudinal distribution reflecting the segmental (metameric) functional specialization of the spinal cord. Normalizing the SUVmax and SUVmean of the spinal cord to the liver radiotracer uptake could be useful in multi-institutional comparisons and studies.

## Supplementary information

**Additional file 1: Table S1.** Median, 1^st^ and 3^rd^ quartile of SUVmax, SUVmean and NSUVmean values measured in spinal cord in both PET-CT and PET-MR, with 3mm ROI and 9mm VOI. **Table S2.** Median, 1^st^ and 3^rd^ quartile of SUVmean and NSUVmean values measured in bone marrow in both PET-CT and PET-MR, with 3mm ROI and 9mm VOI. **Table S3.** Median and 1^st^ and 3^rd^ quartile of the average liver values calculated for each method and ROI/VOI. **Table S4.** Median and 1^st^ and 3^rd^ quartile of SUVmax values measured in spinal cord in both PET-CT and PET-MR, with 3mm ROI and 9mm VOI, between the two subgroups, without and with increased uptake at visual examination in the cervical enlargement (Group 1 and Group 2 respectively).

## Data Availability

The datasets generated and analyzed during the current study are available from the corresponding author on reasonable request.
