# Supplementary material for: [18F]FDG uptake of the normal spinal cord in PET/MR imaging: comparison with PET/CT imaging
Source: EJNMMI Res. 2020 Aug 6;10:91. doi: 10.1186/s13550-020-00680-8 (PMC7410944; doi:10.1186/s13550-020-00680-8)
Supplement: Supplementary file 1 — Additional file 1: Table S1. Median, 1st and 3rd quartile of SUVmax, SUVmean and NSUVmean values measured in spinal cord in both PET-CT and PET-MR, with 3mm ROI and 9mm VOI. Table S2. Median, 1st and 3rd quartile of SUVmean and NSUVmean values measured in bone marrow in both PET-CT and PET-MR, with 3mm ROI and 9mm VOI. Table S3. Median and 1st and 3rd quartile of the average liver values calculated for each method and ROI/VOI. Table S4. Median and 1st and 3rd quartile of SUVmax values measured in spinal cord in both PET-CT and PET-MR, with 3mm ROI and 9mm VOI, between the two subgroups, without and with increased uptake at visual examination in the cervical enlargement (Group 1 and Group 2 respectively). [file 13550_2020_680_MOESM1_ESM.docx]

**Table S1** Median, 1^st^ and 3^rd^ quartile of SUVmax, SUVmean and NSUVmean values measured in spinal cord in both PET-CT and PET-MR, with 3mm ROI and 9mm VOI.

| **Spinal** | **SUVmax in 3mm ROI** | | | | | | | **SUVmean in 3mm ROI** | | | | | | | **NSUVmean in 3mm ROI** | | | | | | |
| --- | --- | --- | --- | --- | --- | --- | --- | --- | --- | --- | --- | --- | --- | --- | --- | --- | --- | --- | --- | --- | --- |
| **Cord** | PET-CT | | | PET-MR | | | p | PET-CT | | | PET-MR | | | p | PET-CT | | | PET-MR | | | p |
|  | q1 | median | q3 | q1 | median | q3 | Wilcoxon | q1 | median | q3 | q1 | median | q3 | Wilcoxon | q1 | median | q3 | q1 | median | q3 | Wilcoxon |
| **C2** | 1.50 | 1.85 | 2.12 | 1.61 | 1.83 | 2.24 | 4.60E-01 | 1.34 | 1.58 | 1.92 | 1.33 | 1.55 | 2.00 | 5.38E-01 | 0.69 | 0.77 | 1.11 | 0.89 | 1.11 | 1.39 | 3.71E-01 |
| **C5** | 1.56 | 1.93 | 2.27 | 1.80 | 2.13 | 2.53 | 4.00E-03 | 1.44 | 1.73 | 2.05 | 1.55 | 1.86 | 2.21 | 5.50E-02 | 0.67 | 0.88 | 1.14 | 1.04 | 1.27 | 1.57 | 2.23E-01 |
| **T6** | 1.32 | 1.52 | 1.77 | 1.34 | 1.52 | 1.83 | 4.88E-01 | 1.21 | 1.31 | 1.53 | 1.16 | 1.37 | 1.54 | 7.02E-01 | 0.56 | 0.73 | 1.20 | 0.76 | 0.93 | 1.13 | 2.76E-01 |
| **T12** | 1.61 | 1.88 | 2.23 | 1.36 | 1.79 | 2.04 | 3.00E-01 | 1.5 | 1.69 | 1.97 | 1.18 | 1.62 | 1.80 | 1.72E-01 | 0.66 | 0.94 | 1.63 | 0.88 | 1.13 | 1.34 | 8.16E-01 |
| **CSF L3** | 0.94 | 1.13 | 1.48 | 0.75 | 0.89 | 1.17 | 1.10E-02 | 0.81 | 0.96 | 1.25 | 0.55 | 0.79 | 1.03 | 1.10E-02 | 0.39 | 0.57 | 0.83 | 0.41 | 0.55 | 0.67 | 2.88E-01 |
|  | **SUVmax in 9mm VOI** | | | | | | | **SUVmean in 9mm VOI** | | | | | | | **NSUVmean in 9mm VOI** | | | | | | |
|  | PET-CT | | | PET-MR | | | p | PET-CT | | | PET-MR | | | p | PET-CT | | | PET-MR | | | p |
|  | q1 | median | q3 | q1 | median | q3 | Wilcoxon | q1 | median | q3 | q1 | median | q3 | Wilcoxon | q1 | median | q3 | q1 | median | q3 | Wilcoxon |
| **C2** | 1.93 | 2.32 | 2.51 | 1.88 | 2.17 | 2.54 | 6.32E-01 | 1.31 | 1.55 | 1.83 | 1.18 | 1.32 | 1.6 | 1.34E-03 | 0.66 | 0.74 | 1.33 | 0.77 | 0.90 | 1.00 | 9.28E-01 |
| **C5** | 1.86 | 2.18 | 2.48 | 2.24 | 2.42 | 2.90 | 6.91E-05 | 1.28 | 1.54 | 1.79 | 1.36 | 1.50 | 1.63 | 7.32E-01 | 0.64 | 0.78 | 1.04 | 0.87 | 1.06 | 1.17 | 5.86E-01 |
| **T6** | 1.86 | 2.08 | 2.51 | 1.98 | 2.26 | 2.87 | 1.90E-02 | 1.21 | 1.39 | 1.54 | 1.15 | 1.27 | 1.48 | 5.43E-02 | 0.57 | 0.73 | 1.33 | 0.74 | 0.82 | 0.94 | 5.95E-01 |
| **T12** | 1.96 | 2.32 | 2.83 | 1.78 | 2.11 | 2.46 | 3.64E-02 | 1.41 | 1.63 | 1.84 | 1.04 | 1.34 | 1.58 | 1.33E-05 | 0.68 | 0.81 | 1.25 | 0.68 | 0.88 | 1.10 | 5.42E-01 |
| **CSF L3** | 1.60 | 1.82 | 2.15 | 1.20 | 1.37 | 1.65 | 2.39E-04 | 0.95 | 1.09 | 1.23 | 0.7 | 0.82 | 1.01 | 2.80E-06 | 0.47 | 0.58 | 1.06 | 0.46 | 0.54 | 0.64 | 5.10E-02 |

**Table S2** Median, 1^st^ and 3^rd^ quartile of SUVmean and NSUVmean values measured in bone marrow in both PET-CT and PET-MR, with 3mm ROI and 9mm VOI.

| **Bone** | **SUVmean in 3mm ROI** | | | | | | | **NSUVmean in 3mm ROI** | | | | | | |
| --- | --- | --- | --- | --- | --- | --- | --- | --- | --- | --- | --- | --- | --- | --- |
| **Marrow** | PET-CT | | | PET-MR | | | p | PET-CT | | | PET-MR | | | p |
|  | q1 | median | q3 | q1 | median | q3 | Wilcoxon | q1 | median | q3 | q1 | median | q3 | Wilcoxon |
| **C2** | 1.25 | 1.47 | 2.01 | 1.46 | 1.98 | 2.66 | 7.10E-06 | 0.56 | 0.69 | 1.18 | 0.88 | 1.30 | 1.88 | 1.12E-01 |
| **C5** | 1.3 | 1.56 | 1.97 | 1.69 | 2.06 | 2.54 | 3.57E-05 | 0.58 | 0.80 | 1.44 | 1.11 | 1.47 | 1.72 | 8.80E-02 |
| **T6** | 1.74 | 2.29 | 2.92 | 2.08 | 2.58 | 3.32 | 4.18E-03 | 0.90 | 1.20 | 1.96 | 1.31 | 1.69 | 2.47 | 1.77E-01 |
| **T12** | 1.72 | 2.32 | 2.97 | 1.87 | 2.21 | 3.12 | 7.31E-01 | 0.83 | 1.21 | 2.16 | 1.17 | 1.48 | 2.33 | 1.76E-01 |
| **L3** | 1.8 | 2.22 | 2.78 | 1.91 | 2.49 | 3.24 | 9.37E-03 | 0.87 | 1.23 | 1.89 | 1.10 | 1.56 | 2.43 | 2.41E-01 |
|  | **SUVmean in 9mm VOI** | | | | | | | **NSUVmean in 9mm VOI** | | | | | | |
|  | PET-CT | | | PET-MR | | | p | PET-CT | | | PET-MR | | | p |
|  | q1 | median | q3 | q1 | median | q3 | Wilcoxon | q1 | median | q3 | q1 | median | q3 | Wilcoxon |
| **C2** | 1.2 | 1.38 | 1.81 | 1.31 | 1.57 | 1.99 | 5.44E-03 | 0.53 | 0.69 | 1.57 | 0.79 | 0.93 | 1.25 | 3.75E-01 |
| **C5** | 1.23 | 1.56 | 1.84 | 1.55 | 1.75 | 2.14 | 2.68E-05 | 0.59 | 0.78 | 1.42 | 0.88 | 1.10 | 1.41 | 3.41E-01 |
| **T6** | 1.61 | 2.15 | 2.49 | 1.92 | 2.24 | 2.92 | 9.50E-04 | 0.78 | 1.03 | 2.16 | 1.15 | 1.38 | 1.84 | 3.28E-01 |
| **T12** | 1.75 | 2.17 | 2.77 | 1.8 | 2.09 | 2.75 | 2.26E-01 | 0.87 | 1.14 | 2.68 | 1.03 | 1.28 | 1.66 | 4.41E-01 |
| **L3** | 1.79 | 2.19 | 2.8 | 1.89 | 2.24 | 2.76 | 2.90E-02 | 0.83 | 1.14 | 2.39 | 1.09 | 1.52 | 1.79 | 4.11E-01 |

**Table S3** Median and 1^st^ and 3^rd^ quartile of the average liver values calculated for each method and ROI/VOI.

|  |  | **ROI = 3mm** |  |  | **VOI = 9mm** |  |
| --- | --- | --- | --- | --- | --- | --- |
| **PET-MR** | q1 | **Mean liver** | q3 | q1 | **Mean liver** | q3 |
| SUV_mean | 1.24 | 1.60 | 1.82 | 1.28 | 1.65 | 1.72 |
| SUV_max | 1.34 | 1.78 | 1.93 | 1.70 | 2.21 | 2.38 |
| **PET-CT** |  |  |  |  |  |  |
| SUV_mean | 1.21 | 1.79 | 2.22 | 1.26 | 1.77 | 2.17 |
| SUV_max | 2.00 | 2.36 | 2.53 | 2.26 | 2.76 | 2.92 |

**Table S4** Median and 1^st^ and 3^rd^ quartile of SUVmax values measured in spinal cord in both PET-CT and PET-MR, with 3mm ROI and 9mm VOI, between the two subgroups, without and with increased uptake at visual examination in the cervical enlargement (Group 1 and Group 2 respectively).

| **Group 1** | **SUVmax in 3mm ROI** | | | | | | | | | | | | **SUVmax in 9mm VOI** | | | | | | | | | | | | |
| --- | --- | --- | --- | --- | --- | --- | --- | --- | --- | --- | --- | --- | --- | --- | --- | --- | --- | --- | --- | --- | --- | --- | --- | --- | --- |
| **Spinal** | PET-CT | | | | | PET-MR | | | | | p | | PET-CT | | | | | PET-MR | | | | | | p | |
| **Cord** | q1 | | median | | q3 | q1 | | median | | q3 | Wilcoxon | | q1 | | median | | q3 | q1 | | median | | q3 | | Wilcoxon |  |
| **C2** | 1.34 | | 1.52 | | 1.73 | 1.45 | | 1.68 | | 1.85 | 4.84E-02 | | 1.54 | | 1.83 | | 2.30 | 1.73 | | 1.89 | | 2.03 | | 7.56E-01 |  |
| **C5** | 1.35 | | 1.55 | | 1.68 | 1.69 | | 2.03 | | 2.32 | 1.05E-04 | | 1.63 | | 1.86 | | 2.09 | 2.02 | | 2.32 | | 2.42 | | 1.34E-04 |  |
| **T6** | 1.22 | | 1.43 | | 1.71 | 1.26 | | 1.40 | | 1.51 | 9.85E-01 | | 1.71 | | 1.95 | | 2.42 | 1.83 | | 1.97 | | 2.67 | | 4.09E-01 |  |
| **T12** | 1.37 | | 1.70 | | 2.17 | 1.31 | | 1.49 | | 1.76 | 1.53E-02 | | 1.88 | | 2.19 | | 2.78 | 1.46 | | 1.87 | | 2.20 | | 1.00E-04 |  |
| **CSF L3** | 0.94 | | 1.12 | | 1.32 | 0.62 | | 0.75 | | 0.90 | 1.92E-02 | | 1.56 | | 1.71 | | 2.09 | 1.04 | | 1.24 | | 1.37 | | 1.00E-04 |  |
| **Group 2** | **SUVmax in 3mm ROI** | | | | | | | | | | | **SUVmax in 9mm VOI** | | | | | | | | | | | | | |
| **Spinal** | PET-CT | | | | | PET-MR | | | | | p | PET-CT | | | | | | PET-MR | | | | | p | | |
| **Cord** | q1 | median | | q3 | | q1 | median | | q3 | | Wilcoxon | q1 | | median | | q3 | | q1 | median | | q3 | | Wilcoxon | |  |
| **C2** | 1.94 | 2.11 | | 2.58 | | 1.65 | 2.20 | | 2.43 | | 6.09E-01 | 2.39 | | 2.48 | | 2.74 | | 2.21 | 2.45 | | 2.81 | | 1.27E-02 | |  |
| **C5** | 2.09 | 2.27 | | 2.47 | | 2.01 | 2.53 | | 2.75 | | 4.12E-01 | 2.37 | | 2.48 | | 3.04 | | 2.42 | 2.90 | | 3.32 | | 2.90E-02 | |  |
| **T6** | 1.46 | 1.56 | | 2.05 | | 1.52 | 1.75 | | 2.17 | | 3.20E-01 | 2.03 | | 2.42 | | 2.51 | | 2.16 | 2.49 | | 3.25 | | 1.75E-02 | |  |
| **T12** | 1.67 | 2.02 | | 2.34 | | 1.79 | 2.04 | | 2.33 | | 3.74E-01 | 2.04 | | 2.44 | | 2.93 | | 1.99 | 2.46 | | 2.75 | | 8.92E-01 | |  |
| **CSF L3** | 0.94 | 1.16 | | 1.55 | | 0.87 | 1.12 | | 1.34 | | 5.17E-01 | 1.65 | | 1.99 | | 2.18 | | 1.38 | 1.54 | | 2.20 | | 1.91E-01 | |  |
